# Supplementary material for: Imputation of plasma lipid species to facilitate integration of lipidomic datasets
Source: Nat Commun. 2024 Feb 20;15:1540. doi: 10.1038/s41467-024-45838-3 (PMC10879118; doi:10.1038/s41467-024-45838-3)
Supplement: Supplementary file 1 — Supplementary Information [file 41467_2024_45838_MOESM1_ESM.pdf]

Imputation of plasma lipid species to facilitate integration of lipidomic datasets

Aleksandar Dakic[1], Jingqin Wu[1], Tingting Wang[1], Kevin Huynh[1,2,3], Natalie Mellett[1], Thy Duong[1], Habtamu B Beyene[1,2], Dianna J Magliano[1], Jonathan E Shaw[1], Melinda J Carrington[1,3], Mike Inouye[1], Jean Y Yang [4,5], Gemma A Figtree [6,7], Joanne E. Curran[8], John Blangero[8], John Simes[9], the LIPID Study Investigators[#], Corey Giles[1,2,3*], Peter J Meikle[1,2,3,10*]

[1]Baker Heart and Diabetes Institute, Melbourne, Victoria, Australia.
[2]Baker Department of Cardiovascular Research, Translation and Implementation, La Trobe University, Melbourne, VIC, 3086, Australia.
[3]Baker Department of Cardiometabolic Health, The University of Melbourne, VIC, 3010, Australia.
[4]School of Mathematics and Statistics, The University of Sydney, Camperdown, NSW 2006, Australia
[5]Charles Perkins Centre, The University of Sydney, Camperdown, NSW 2006, Australia
[6]Kolling Institute of Medical Research, The University of Sydney, St Leonards, NSW 2065, Australia
[7]Department of Cardiology, Royal North Shore Hospital, St Leonards, NSW 2065, Australia
[8]Department of Human Genetics and South Texas Diabetes and Obesity Institute, School of Medicine at University of Texas Rio Grande Valley, Brownsville, TX, United States.
[9]National Health and Medical Research Council of Australia (NHMRC) Clinical Trials Centre, University of Sydney, Sydney, New South Wales, Australia.
[10]Department of Diabetes, Central Clinical School, Monash University, Clayton, VIC, 3800, Australia
[#]A list of authors and their affiliations appears at the end of the paper.

*These authors jointly supervised this work

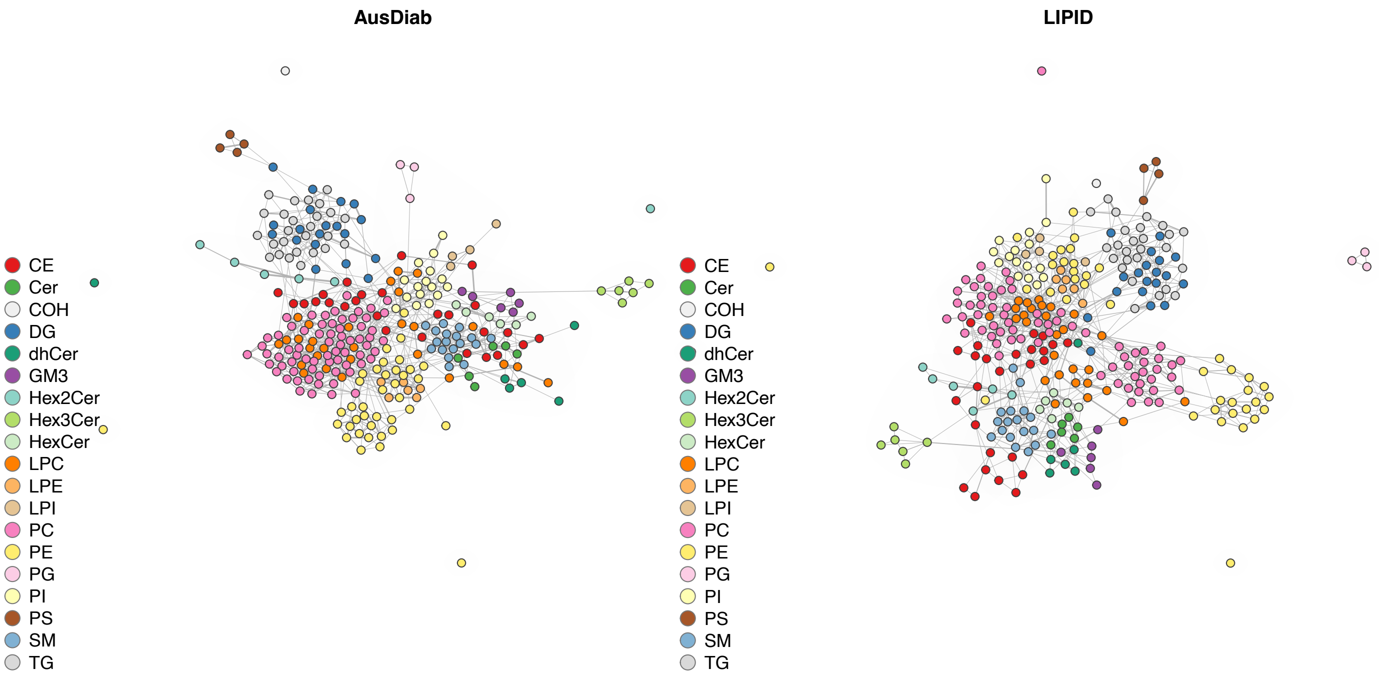

Supplementary Figure 1. Partial correlation networks between lipid species in AusDiab and LIPID studies, presented as Gaussian graphical models after Louvain community detection. Only lipids measured in both data sets were included in the analysis and the 1000 strongest associations are shown for each data set.

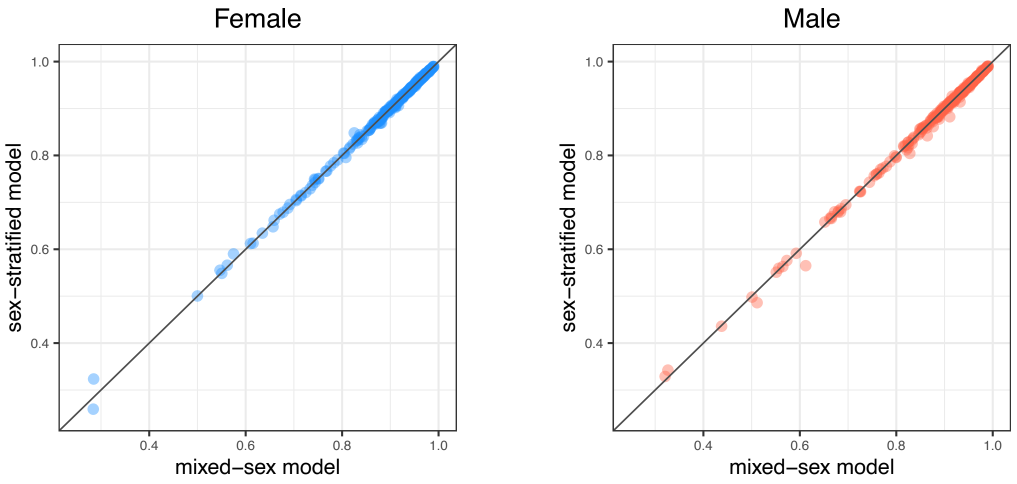

Supplementary Figure 2. Stratification on sex does not improve accuracy of the lipid prediction models. The accuracy of lipid predictions obtained from the sex-stratified and mixed sex models (results of the latter being split into sexes to enable comparison). The analysis focused on lipids measured in both the AusDiab and LIPID study (matching lipids). Predictive models for individual lipids were build using the remaining matching lipids in AusDiab data. The models were used to predict corresponding individual lipids in the LIPID study and accuracy of their prediction was assessed as the correlation between predicted and measured (conceptually masked) lipid concentrations in the LIPID study. Each dot represents prediction accuracy for one of 294 lipid species. The line marks an equivalent prediction accuracy in the two types of models.

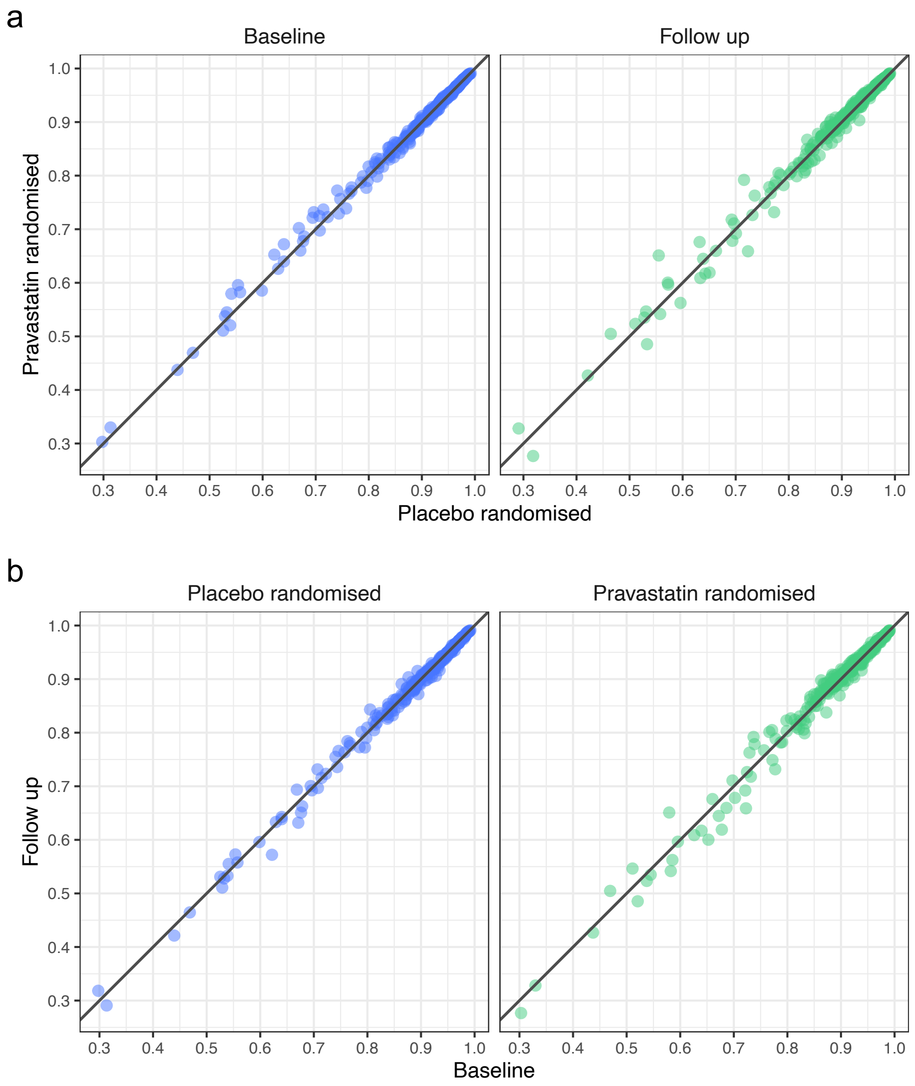

Supplementary Figure 3. Transferability of predictive models despite differential use of lipid lowering treatments in the AusDiab and LIPID study. Predictive models built using only lipid species as predictors (age, sex, BMI and lipid lowering treatment predictors removed). a The accuracy of lipid predictions in the LIPID study between placebo and pravastatin randomised patient groups, at baseline (when neither group received treatment) and follow up (when only pravastatin group was exposed to the treatment). Each dot represents prediction accuracy for one of 294 lipid species, assessed as the correlation between predicted and measured (conceptually masked) lipid concentrations within a given treatment group and time point subset. Blue dots indicate no exposure to pravastatin in either compared subsets, green dots indicate pravastatin exposure in one of the two subsets. The line marks an equivalent prediction accuracy in two given subsets. Only lipids measured in both data sets were included in the analysis, and 13 most discordant lipids were excluded from the models. **b** Comparing the accuracy of lipid predictions in the LIPID study between baseline and follow up, in placebo randomised (neither timepoint received treatment) and pravastatin randomised patient group (only follow up subset was exposed to the treatment).

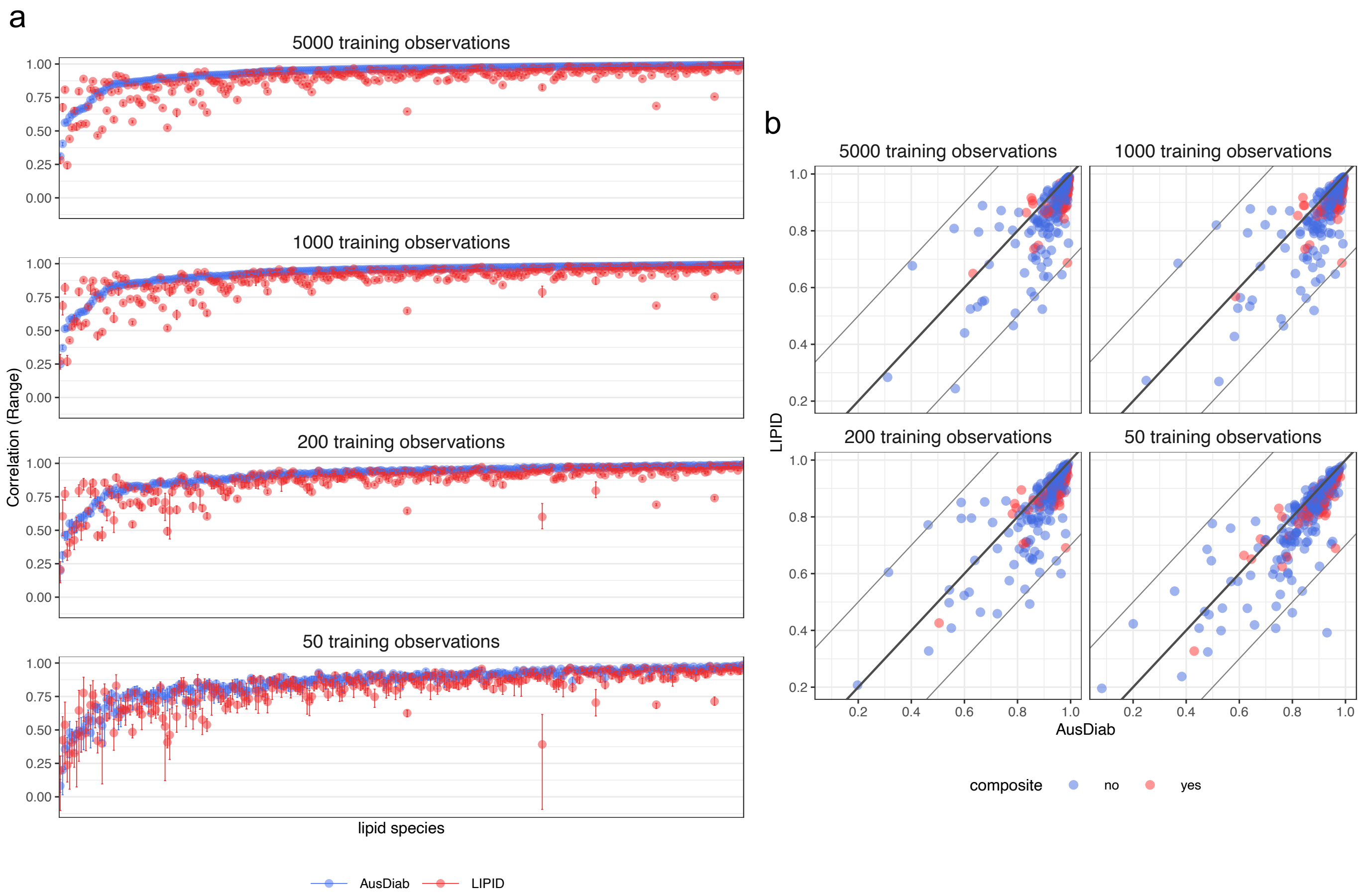

Supplementary Figure 4. Transferability of lipid prediction models from the Ausdiab to LIPID study - the effect of reducing training sample size. a The accuracy of lipid predictions, measured as the correlation between observed and predicted concentrations, in AusDiab (blue) and LIPID (red). The mean and range of correlations are shown, across n=10 predictions for each individual lipid based on random subsamples of training observations. Facet labels indicate the number of observations used to build models in the AusDiab. Only lipids measured in both data sets were included in the analysis. AusDiab predictions were assessed on a hold-out set (n=1986 observations); LIPID predictions were assessed on all baseline observations (n=5991). **b** The average accuracy of lipid predictions in AusDiab and LIPID, across 10 random subsamples of training observations for each target lipid. Individual lipid species (blue) and composite lipids (red). The line of an equivalent prediction accuracy for a lipid in AusDiab and LIPID is drawn, as well as the departure of 0.3, in correlation.

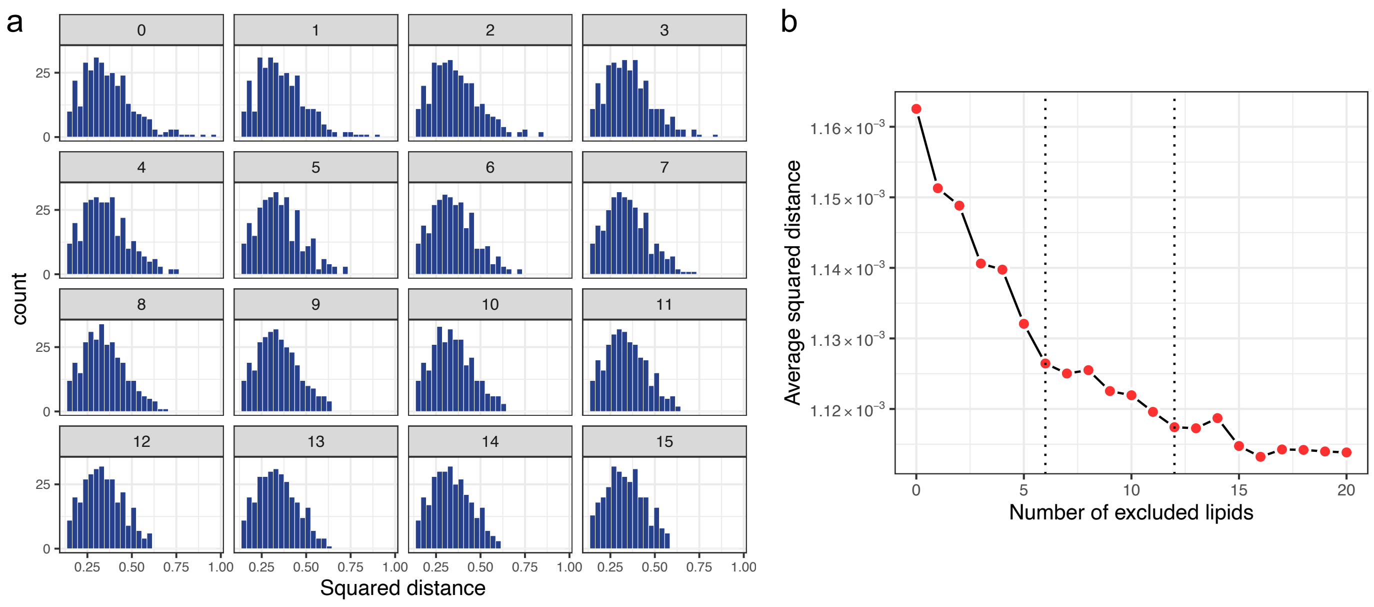

Supplementary Figure 5. Identifying the discordant lipid species between the AusDiab and SAFHS studies. a The distribution of the squared Euclidean distance between partial correlation vectors of corresponding lipid species in AusDiab and SAFHS—a measure of discordance between identical lipid species in the two data sets. After each calculation, the most discordant lipid was removed from both data sets and partial correlation matrices as well as distances between corresponding lipids recalculated. Facet labels indicate the number of lipids removed at the time. **b** The average squared distance between the remaining pairs of corresponding lipid species in AusDiab and SAFHS after the removal of the most discordant lipid at each point.

Supplementary Table 1. Anthropometric and clinical characteristics of the AusDiab and LIPID study

characteristic	AusDiab	LIPID
n	10339	5991
Age [years][a]	51.32 (14.27)	62.59 (8.38)
Sex, n (% female)	5685 (55.0)	1022 (17.1)
BMI [kg/m2] [a]	26.95 (4.94)	26.81 (3.91)
Total cholesterol [mmol/L] [a]	5.66 (1.07)	5.66 (0.82)
HDL cholesterol [mmol/L] [a]	1.43 (0.38)	0.95 (0.24)
LDL cholesterol [mmol/L] [a]	3.54 (0.94)	3.89 (0.75)
Triglycerides [mmol/L] [a]	1.54 (1.07)	1.81 (0.92)
Lipid-lowering treatment, n (%)[*]	871 (8.4)	2989 (0*)
Diabetes, n (%)	686 (6.6)	511 (8.5)
Previous cardiovascular event, n (%)[#]	845 (8.2)	5991 (100.0)
Incident cardiovascular event, n (%)[#]	590 (5.7)	1359 (22.7)

[a]Values for continuous variables are expressed as mean (SD).

[*]Lipid-lowering treatment in the AusDiab cohort was derived from the question “are you currently taking tablets to lower your cholesterol/triglycerides?” and relates to any lipid-lowering medication taken at baseline – when samples were taken for the lipidomic analysis. In the LIPID trial, patients were not taking pravastatin or any other lipid-lowering medication at the baseline, as a part of run-in phase. 49.9% of patients were later randomised to pravastatin treatment (40mg), after the baseline samples were taken for lipidomic analysis. The follow up samples were taken 12 months after the randomisation.

[#]Previous cardiovascular event in the AusDiab cohort was based on combining responses to questions “have you been told you have had a heart attack / stroke / angina?”. All the patients involved in the LIPID trial had a cardiovascular event as the main criterion for recruitment (acute myocardial infarction (AMI) or unstable angina pectoris (UAP)). Consequently, the Incident cardiovascular event category in the LIPID trial refers solely to the subsequent, secondary events (nonfatal MI, nonfatal stroke, or cardiovascular death).

For the purpose of this work, we treated the missing values and no responses in the categorical variables above as negative responses.

Supplementary Table 2. Lipid species excluded from the predictor set due to large discordance between their distributions in the two data sets.

Lipid species excluded from the predictor set
LPC(O-20:1)
TG(54:0) [NL-18:0]
PC(38:2)
PC(O-34:2)
DG(16:0_16:0)
PC(P-18:0/22:6)
SM(d18:2/20:0)
SM(d18:1/20:0)/SM(d16:1/22:0)
PC(O-40:7)
LPC(O-18:0)
PE(O-18:0/22:6)
LPC(O-18:1)
SM(37:2)

Supplementary Table 3. Lipid species not predicted in the target data (the LIPID study) due to poor prediction accuracy achieved in the reference data (the AusDiab study).

Lipid	Best correlation
Sph(d16:1)	0.137
Cer(d20:1/26:0)	0.226
Cer(d18:2/17:0)	0.237
SHexCer(d18:2/18:0(OH))	0.286
DE(16:0)	0.344
GM3(d20:1/18:0)	0.364
SHexCer(d18:2/18:0)	0.37
SHexCer(d18:1/18:0(OH))	0.375
Sph(d18:2)	0.375
SHexCer(d18:1/18:0)	0.407
Cer1P(d18:1/16:0)	0.516
Cer(d18:2/14:0)	0.52
GM3(d18:2/24:1)	0.529
SHexCer(d18:1/24:0(OH))	0.536
SHexCer(d18:1/24:0)	0.54
PS(38:5)	0.549
Cer(d19:1/16:0)	0.55
Cer(d18:1/14:0)	0.554
S1P(d18:0)	0.557
Sph(d17:1)	0.569
HexCer(d18:2/18:0)	0.588
Cer(d19:1/26:0)	0.592
Hex2Cer(d16:1/24:1)	0.594
SHexCer(d18:1/24:1)	0.595
S1P(d18:2)	0.599
Sph(d18:1)	0.599
