# Supplementary material for: Imputation of plasma lipid species to facilitate integration of lipidomic datasets
Source: Nat Commun. 2024 Feb 20;15:1540. doi: 10.1038/s41467-024-45838-3 (PMC10879118; doi:10.1038/s41467-024-45838-3)
Supplement: Supplementary file 3 — Description of Additional Supplementary Files [file 41467_2024_45838_MOESM3_ESM.pdf]

Description of Additional Supplementary Files

Supplementary Data 1. LIPID to AusDiab lipid species mapping

Supplementary Data 2. AusDiab to LIPID lipid species mapping.

Supplementary Data 3. MRM transitions and conditions for examined lipid species.

Supplementary Data 4. Conditions for tandem mass spectrometry analysis of the LIPID study.
