# Supplementary material for: Imputation of plasma lipid species to facilitate integration of lipidomic datasets
Source: Nat Commun. 2024 Feb 20;15:1540. doi: 10.1038/s41467-024-45838-3 (PMC10879118; doi:10.1038/s41467-024-45838-3)
Supplement: Supplementary file 8 — Reporting Summary [file 41467_2024_45838_MOESM8_ESM.pdf]

nature portfolio

Corresponding author(s): Peter J Meikle
Corey Giles

Last updated by author(s): Dec 19, 2023

Reporting Summary

Nature Portfolio wishes to improve the reproducibility of the work that we publish. This form provides structure for consistency and transparency in reporting. For further information on Nature Portfolio policies, see our Editorial Policies and the Editorial Policy Checklist.

Statistics

For all statistical analyses, confirm that the following items are present in the figure legend, table legend, main text, or Methods section.

n/a	Confirmed	
☐	☒	The exact sample size (*n*) for each experimental group/condition, given as a discrete number and unit of measurement
☐	☒	A statement on whether measurements were taken from distinct samples or whether the same sample was measured repeatedly
☐	☒	The statistical test(s) used AND whether they are one- or two-sided<br>*Only common tests should be described solely by name; describe more complex techniques in the Methods section.*
☐	☒	A description of all covariates tested
☐	☒	A description of any assumptions or corrections, such as tests of normality and adjustment for multiple comparisons
☐	☒	A full description of the statistical parameters including central tendency (e.g. means) or other basic estimates (e.g. regression coefficient) AND variation (e.g. standard deviation) or associated estimates of uncertainty (e.g. confidence intervals)
☐	☒	For null hypothesis testing, the test statistic (e.g. *F*, *t*, *r*) with confidence intervals, effect sizes, degrees of freedom and *P* value noted<br>*Give P values as exact values whenever suitable.*
☒	☐	For Bayesian analysis, information on the choice of priors and Markov chain Monte Carlo settings
☒	☐	For hierarchical and complex designs, identification of the appropriate level for tests and full reporting of outcomes
☐	☒	Estimates of effect sizes (e.g. Cohen's *d*, Pearson's *r*), indicating how they were calculated

Our web collection on statistics for biologists contains articles on many of the points above.

Software and code

Policy information about availability of computer code

Data collection	Mass Hunter (Agilent Technologies) software was used for HPLC-MS/MS data acquisition; version 8.0 for the LIPID, and version 9.0 for the AusDiab and SAFHS studies.
Data analysis	Data analysis was performed on open-source software R (version 4.2.1). All predictive models were fit using R package glmnet (https://cran.r-project.org/web/packages/glmnet/index.html). Code to reproduce all analyses has been made freely available on github: https://github.com/BakerMetabolomics/LIPID_imputation

For manuscripts utilizing custom algorithms or software that are central to the research but not yet described in published literature, software must be made available to editors and reviewers. We strongly encourage code deposition in a community repository (e.g. GitHub). See the Nature Portfolio guidelines for submitting code & software for further information.

Data

Policy information about availability of data

All manuscripts must include a data availability statement. This statement should provide the following information, where applicable:

- Accession codes, unique identifiers, or web links for publicly available datasets
- A description of any restrictions on data availability
- For clinical datasets or third party data, please ensure that the statement adheres to our policy

Because of the participant consent obtained as part of the recruitment process for the AusDiab and LIPID studies, it is not possible to make data publicly available

(including the individual deidentified data). Individual-level AusDiab data are available for analyses that do not conflict with ongoing studies, through application to the study lead Professor Jonathan Shaw and the AusDiab Study Committee (Email: Jonathan.Shaw@baker.edu.au). The timeframe for response to such requests is within two months.

Individual-level LIPID data are available for analyses that do not conflict with ongoing studies, through application to the study lead Professor John Simes and the LIPID Study Investigators (Email: John.Simes@sydney.edu.au). The timeframe for response to such requests is within two months.

The SAFHS anthropometric and genomic data are publicly available through dbGaP (accession numbers: phs001215.v4.p2 [https://www.ncbi.nlm.nih.gov/projects/gap/cgi-bin/study.cgi?study_id=phs001215.v4.p2], phs000847.v2.p1 [https://www.ncbi.nlm.nih.gov/projects/gap/cgi-bin/study.cgi?study_id=phs000847.v2.p1], phs000462.v2.p1 [https://www.ncbi.nlm.nih.gov/projects/gap/cgi-bin/study.cgi?study_id=phs000462.v2.p1]), whereas lipidomic data are available from J.E Curran, Email: joanne.curran@utrgv.edu via a material transfer agreement for work consistent with the informed consent.

The summary statistics for the AusDiab and LIPID studies are provided in the Supplementary files. Data generated in this study are provided in the Source Data file.

Research involving human participants, their data, or biological material

Policy information about studies with human participants or human data. See also policy information about sex, gender (identity/presentation), and sexual orientation and race, ethnicity and racism.

Reporting on sex and gender

We followed the sex and gender equity in research (SAGER) guideline and included the recommended information in our study. Sex information was based on self-reported questionnaires. Overall, AusDiab cohort consisted of 5685 (55%) females and 4654 (45%) males; LIPID trial captured predominantly male population with 1022 (17.1%) females and 4969 (82.9%) males; SAFHS cohort analysed consisted of 3409 (61%) females and 2181 (39%) males. Sex was included as one of the predictor variables in all models in this study.

Reporting on race, ethnicity, or other socially relevant groupings

SAFHS study consists of Mexican American participants only, whereas the AusDiab and LIPD studies represent samples from Australian population. Ethnic distinction is only used to confirm that imputation models for lipid species can be transfered between these two populations without significant loss of prediction accuracy.

Population characteristics

Population characteristics are described in Supplementary Table 1 of the manuscript. Main characteristics of the two studies used are:
AusDiab study (n=10339): 55% female, mean age 51.32 (SD:14.27), mean MBI 26.95 (SD:4.94), 8.4% taking lipid-lowering treatment, 8.2% had previous cardiovascular event at recruitment, and in 5.7% it occurred post-recruitment.
LIPID study (n=5991): 17.1% female, mean age 62.59 (SD:8.38), mean MBI 26.81 (SD:3.91), 0% taking lipid-lowering treatment at baseline, 100% had previous cardiovascular event as a recruitment prerequisite, and in 22.7% it occurred post-recruitment.
SAFHS study: (n=5590) 61% females, mean age 43.22 (SD:16.19), BMI and lipid-lowering treatment variables were not used in the models to avoid missing data and reducing the sample size

Recruitment

Our study did not directly involved the recruitment of participants. The recruitment for the AusDiab, LIPID and SAFHS studies has been previously described and referenced in this manuscript (AusDiab: Dunstan et al. 2002; LIPID: LIPID Study Group. 1995, 1998; SAHS: Mitchell et al. 1996). As our study is not primarily concerned with parameter estimation, it is unlikely that the recruitment strategies used have any impact on our findings.

Ethics oversight

This study used samples stored in the AusDiab and LIPID biobanks, which was approved by the Alfred Human Research Ethics Committee, AlfredHealth, Melbourne, Australia (project approval numbers, AusDiab: 41/18, LIPID: 85/11 and 376/22). Studies were conducted in accordance with the ethical principles of the Declaration of Helsinki. No participant compensation was provided. Further validation was performed on the San Antonio Family Heart Study (SAFHS), which was reviewed and approved by the Institutional Review Board at the University of Texas Rio Grande Valley (IRB-18-0245, IRB-18-0255 and IRB-18-0406). The participants provided their written informed consent to participate in this study.

Note that full information on the approval of the study protocol must also be provided in the manuscript.

Field-specific reporting

Please select the one below that is the best fit for your research. If you are not sure, read the appropriate sections before making your selection.

☒ Life sciences ☐ Behavioural & social sciences ☐ Ecological, evolutionary & environmental sciences

For a reference copy of the document with all sections, see nature.com/documents/nr-reporting-summary-flat.pdf

Life sciences study design

All studies must disclose on these points even when the disclosure is negative.

Sample size

We performed lipidomics analysis on two large studies (AusDiab n=10339; LIPID n=5991; SAFHS n=5590). Sample size was determined by the availability of biological samples for analysis. Sample sizes were not predetermined because our study was not primarily concerned with parameter estimation or comparison of means and proportions. In instances where this could be relevant, such as testing the association of individual lipid species with incident cardiovascular mortality (incident rate of 22.7%), we can confirm that sample size of 4927 is sufficient to detect an odds ratio increase of only 0.1, with two-tailed type I error of 0.05 (95% CI) and type II error of 0.2 (80% power).

Data exclusions	Only samples that did not have sufficient amount of plasma for lipidomics analysis were excluded from analysis.
Replication	The lipid imputation models were generated using 10-fold cross-valiation, splitting the first dataset into training and testing datasets. Furthermore, we validated models using external validation by imputing lipid species measured in the two independent cohorts.
Randomization	The participant recruitment and data collection for the AusDiab, LIPID and SAFHS cohorts were conducted before the current study. It is important to note that the objectives of the original studies and the current study differ. During data analysis, appropriate adjustments were made by including covariates such as age, sex, and BMI in the models. Additional confounders were also taken into account as deemed necessary for the analysis.
Blinding	Since data collection and data analysis were carried out independently by different researchers, no additional blinding measures were implemented. Data analysts, including statisticians and bioinformaticians, work with de-identified data, ensuring that they are unaware of any specific laboratory, clinical, or lipidomics information. All statistical analyses and graphics are conducted using computer software without any manual interventions.

Reporting for specific materials, systems and methods

We require information from authors about some types of materials, experimental systems and methods used in many studies. Here, indicate whether each material, system or method listed is relevant to your study. If you are not sure if a list item applies to your research, read the appropriate section before selecting a response.

Materials & experimental systems

n/a	Involved in the study
☒	☐ Antibodies
☒	☐ Eukaryotic cell lines
☒	☐ Palaeontology and archaeology
☒	☐ Animals and other organisms
☒	☐ Clinical data
☒	☐ Dual use research of concern
☒	☐ Plants

Methods

n/a	Involved in the study
☒	☐ ChIP-seq
☒	☐ Flow cytometry
☒	☐ MRI-based neuroimaging

Plants

Seed stocks	*Report on the source of all seed stocks or other plant material used. If applicable, state the seed stock centre and catalogue number. If plant specimens were collected from the field, describe the collection location, date and sampling procedures.*
Novel plant genotypes	*Describe the methods by which all novel plant genotypes were produced. This includes those generated by transgenic approaches, gene editing, chemical/radiation-based mutagenesis and hybridization. For transgenic lines, describe the transformation method, the number of independent lines analyzed and the generation upon which experiments were performed. For gene-edited lines, describe the editor used, the endogenous sequence targeted for editing, the targeting guide RNA sequence (if applicable) and how the editor was applied.*
Authentication	*Describe any authentication procedures for each seed stock used or novel genotype generated. Describe any experiments used to assess the effect of a mutation and, where applicable, how potential secondary effects (e.g. second site T-DNA insertions, mosiacism, off-target gene editing) were examined.*
